# Comparison of staged LLIF combined with posterior instrumented fusion with posterior instrumented fusion alone for the treatment of adult degenerative lumbar scoliosis with sagittal imbalance

**DOI:** 10.1186/s12891-023-06340-x

**Published:** 2023-04-03

**Authors:** Oujie Lai, Hao Li, Qixing Chen, Yong Hu, Yunling Chen

**Affiliations:** 1 Department of Spine Surgery, Ningbo No.6 Hospital, Ningbo, Zhejiang People’s Republic of China; 2 Department of Spine Surgery, Second Affiliated Hospital, Zhejiang University School of Medicine, Hangzhou, Zhejiang People’s Republic of China

**Keywords:** Adult degenerative lumbar scoliosis, Lateral lumbar interbody fusion, Stage surgery, Posterior instrumented fusion

## Abstract

**Background:**

To retrospectively compare the clinical and radiological outcomes of staged lateral lumbar interbody fusion (LLIF) combined with posterior instrumented fusion(PIF)with PIF alone for the treatment of adult degenerative lumbar scoliosis (ADLS) with sagittal imbalance.

**Methods:**

ADLS patients with sagittal imbalance underwent corrective surgery were included and divided into staged group (underwent multilevel LLIF in the first-stage and PIF in the second-stage) and control group (PIF alone). The clinical and radiological outcomes were evaluated and compared between the two groups.

**Results:**

Forty-five patients with an average age of 69.7±6.3 years were enrolled, including 25 in the staged group and 20 in the control group. Compared with preoperative values, patients in both groups achieved significant improvement in terms of ODI, VAS back, VAS leg and spinopelvic parameters after surgery, which were maintained well during the follow-up period. Compared with control group, total operative time in the staged group was longer, but the amounts of blood loss and blood transfusion were reduced. The average posterior fixation segments were 6.20±1.78 in the staged group and 8.25±1.16 in the control group (*P*<0.01), respectively. Posterior column osteotomy (PCO) was performed in 9 patients (36%) in the staged group, while PCO and/or pedicle subtraction osteotomy were performed in 15 patients (75%) in the control group (P<0.01). There was no difference in complications between the two groups.

**Conclusion:**

Both surgical strategies were effective for the treatment of ADLS with sagittal imbalance. However, staged treatment was less invasive, which reduced the number of posterior fixation segments and osteotomy requirement.

## Background

Adult degenerative lumbar scoliosis (ADLS) is a common spinal deformity in skeletally mature patients, characterized by a lateral curvature of spine in the coronal plane greater than 10°, asymmetric loss of disc height, lumbar kyphosis, and even global sagittal imbalance [1, 2]. The prevalence of ADLS is about 13–30% among people over 40 years old, and it is on the rise as the aging population increases [3]. With the increasing proportion of elderly individuals and longer life expectancy, the treatment of ADLS is gaining increasing attention because of its important impacts on health-related quality of life.

Surgical treatment is recommended when the initial conservative treatment is unsatisfactory. However, surgical treatment for ADLS is challenging due to a high incidence of complications [4]. Decompression alone or combined with limited instrumented fusion is usually performed in patients presenting primarily with radiculopathy or stenosis [5, 6]. However, for ADLS with sagittal imbalance, the traditional method is to extend the posterior instrumented fusion (PIF) to the thoracic vertebra, or even combined with three-column osteotomy, which is associated with great surgical trauma, high complications, and even death [7].

In recent years, there has been an increasing emphasis on minimally invasive treatment, with the aim of reducing surgical trauma and prompting rapid recovery. Transpsoas lateral lumbar interbody fusion (LLIF), as a minimally invasive surgical technique, allows the placement of large interbody cage on bilaterally lateral margins of the dense apophyseal ring, and subsequently results in indirectly neurological decompression and deformity correction [8–10]. Staged LLIF in combination with PIF has been proposed as a promising surgical strategy for ADLS with sagittal imbalance. However, staged treatment is suspected to be associated with higher complications [11]. In addition, the role of LLIF in sagittal deformity correction for ADLS is still controversial [12]. Thus, the purpose of this retrospective study was to compare clinical and radiological outcomes of staged LLIF in combination with PIF or PIF alone in the treatment of ADLS patients with sagittal imbalance.

## Materials and methods

Institutional review board approval was obtained. In this study, we retrospectively reviewed data of ADLS patients who underwent corrective surgeries in our institution between January 2016 and January 2019. The inclusion criteria included (1) older than 50 years; (2) the apical vertebrae in coronal plane is lower than L1 level; (3) coronal Cobb angle > 20°; (4) pelvic incidence (PI)/lumbar lordosis (LL) mismatch$\ge 20^\circ$;(5) sagittal vertical axis (SVA)$\ge$ 6 cm [13]; (6) conservative treatment for more than 3 months; (7) a minimum follow-up period of 1 year. The exclusion criteria included (1) previous spinal fusion surgery (2) bony fusion of lumbar facet joint; (3) combined with congenital spinal stenosis or severe spondylolisthesis; (4) other types of lumbar scoliosis, including idiopathic scoliosis, congenital scoliosis, iatrogenic deformity, and neuromuscular disease.

According to the different corrective strategies, the included patients were divided into staged group and control group. In the staged group, multilevel LLIFs were performed in the first-stage, and posterior pedicle screw fixation and fusion were performed in the second-stage. In the control group, the patients were managed by posterior pedicle screw fixation and fusion alone in single stage. The advantages and disadvantages of the two different surgical strategies were informed to the patients in detail before surgery, who made the final decision for the treatment. Informed consent was obtained from all patients.

### Surgical procedures

#### Staged group

In the first-stage, lateral incision was usually made from the concave side of the scoliosis with patient positioned in lateral decubitus position after general anesthesia. Through retroperitoneal transpsoas trajectory, the lateral side of indicated disc space was exposed, and discectomy was performed until to the contralateral side. For anterior column realignment (ACR), high speed blur was used to remove the paravertebral bridging osteophyte, and anterior longitudinal ligament and annulus were completely released by using a long-handle scalpel or the surgeon’s method of choice. Polyetheretherketone interbody cages (Sanyou, Shanghai, China) with uniform 18 mm anteroposterior width were used. The lateral and vertical dimensions of the cages depended on the anatomic morphology of disc spaces. Allograft demineralized bone matrix combined with bone marrow aspirate were used as bone graft. The patients were encouraged to walk on the first day after the first-stage surgery.

In the second-stage (usually 5–7 days after the first-stage surgery), the patients underwent posterior pedicle screw fixation and fusion. Laminectomy was performed at the levels with symptomatically neurological compression. Transforaminal lumbar interbody fusion (TLIF) was performed at L5/S1 level for patient with the following conditions:(1) nerve root compression or lumbar canal stenosis; (2) segmental instability or spondylolisthesis; (3) severe degeneration of L5/S1 disc; (4) previous surgical history; (5) more than 15° tilt of L5 vertebral body. Posterior column osteotomy (PCO) was performed in patients with PI/LL mismatch > 30° or thoracolumbar kyphotic deformity according to the radiographic examination after first-stage LLIF.

#### Control group

Posterior pedicle screw fixation and fusion was performed in a standard fashion through posterior midline incision under prone position. Laminectomy was performed at the levels with symptomatically neural elements compression. TLIF was performed at unstable levels. PCO, pedicle subtraction osteotomy (PSO) or a combination was performed in patients with severe rigid sagittal and/or coronal deformities.

### Clinical and radiological evaluation

By reviewing medical records, patients’ data regarding demographics, operative time, blood loss, blood transfusion, postoperative hospital stay, surgical details and complications were collected. For patients in the staged group, the blood loss and operative time was calculated by the sum of those during the first- and second-staged surgery. Postoperative short-term psoas weakness, thigh numbness, and pain were regarded as expected aspects of the recovery process, but were regarded as complications when these deficiencies persist beyond 6 months. Complications were categorized as major and minor according to the definition by Glassman et al. [14].

Clinical and radiological assessments were performed preoperatively, postoperatively and at final follow-up. Back and leg pain was evaluated by Visual Analogue Scale (VAS) ranging from 0 (no pain) to 10 (maximal pain). Functional status was evaluated by Oswestry Disability Index (ODI).

**Fig. 1:**
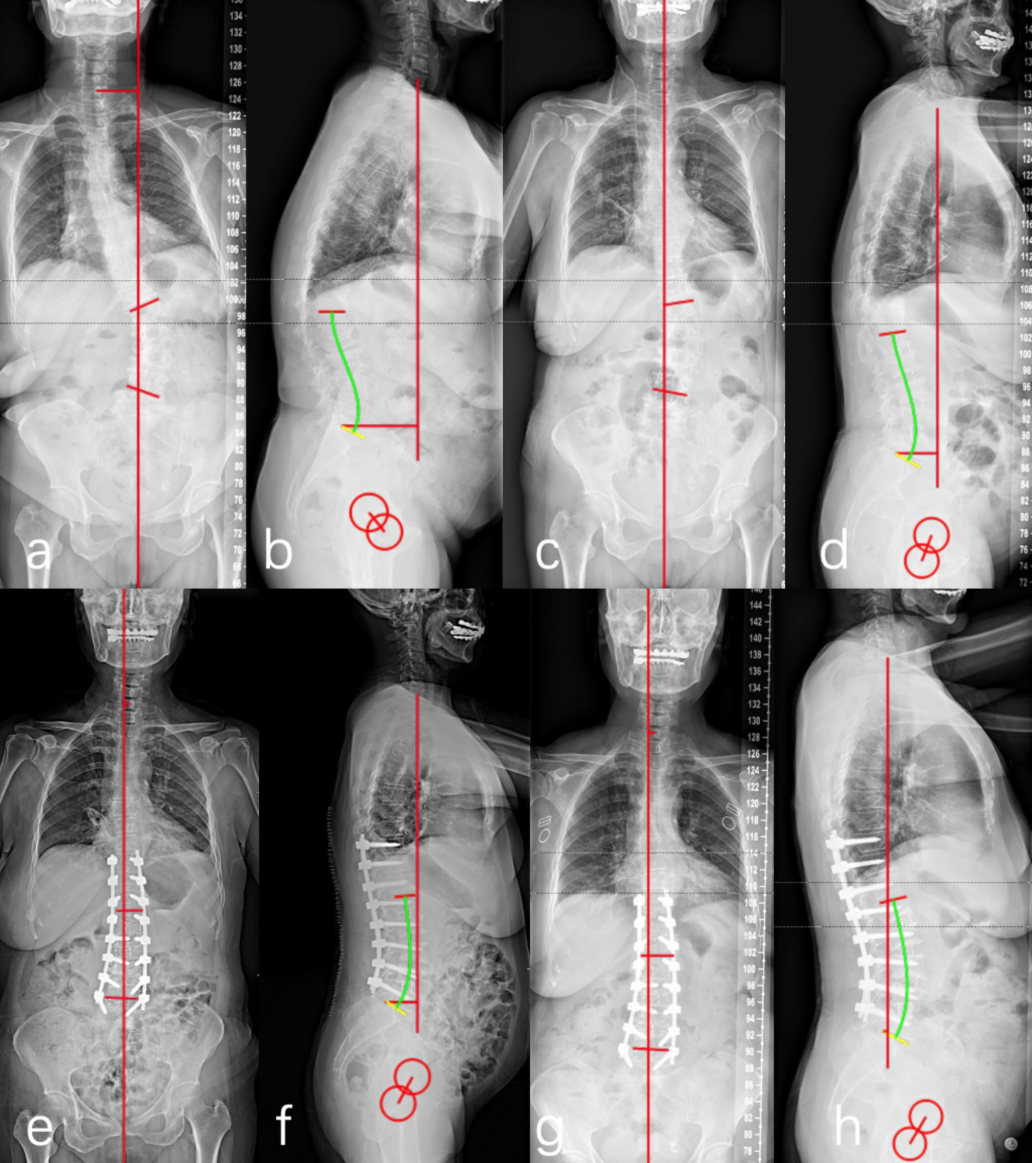
**a,b** Preoperative full-length anteroposterior and lateral radiographs of a 68-year-old female showing severe lumbar scoliosis and sagittal imbalance. **c,d** Coronal and sagittal deformities were partially corrected after the first-stage LLIF from L1/2 to L4/5. **e,f** Coronal and sagittal deformities were further corrected after posterior instrumented fusion from T10-L5. **g,h** The global and regional balance was maintained well at 16 months after surgery

The following spinopelvic parameters were evaluated by using full-length anteroposterior and lateral radiographs: Cobb angle, coronal balance distance (CBD), SVA, LL, PI, pelvic tilt (PT) and PI/LL mismatch (Fig. 1). Proximal junctional kyphosis (PJK) was evaluated during the follow-up period, which was defined as kyphosis increase > 10º between the most upper instrumented vertebra and the vertebra two levels above [15].

### Statistical analysis

SPSS software (version 20.0; Chicago, IL, USA) was used for statistical analysis. Continuous variables were expressed as means$\pm$standard deviation, while qualitative variables were expressed as percentage. Continuous variables were compared by Student’s t-test or Mann-Whiney. Pearson’s Chi-squared test or Fisher’s exact test was used to compared qualitative variables. A result was considered to be statistically significant with *p*-value<0.05.

## Results

A total of 45 patients were enrolled in this study with an average age of 69.71±6.29 years, including 25 in the staged group and 20 in the control group. The average follow-up duration was 26.07±6.64 months. The two groups were similar in terms of gender, age, body mass index (BMI), BMD, symptomatic duration, and follow-up duration (Table 1). Before surgery, all the patients showed back pain. There were 15 patients in the staged group and 13 patients in the control group showing lower extremity radicular pain or claudication (*P*>0.05), respectively.

**Table 1:** Baseline demographic and clinical characteristics of staged and control groups

	Staged group	Control group	P value
Gender(F/M)	19/6	15/5	0.94
Age(yea)	69.4±7.17	70.1±5.13	0.72
BMI(kg/m^2^)	22.81±2.05	23.08±1.73	0.64
BMD	-1.77±0.58	-1.78±0.39	0.98
Symptomatic duration (months)	36.44±10.39	35.25±8.09	0.68
Follow-up duration (months)	24.84±6.69	27.6±6.4	0.17

F Female, M Male

### Surgical results

For the staged group, 83 levels of LLIF were performed in the first-stage with an average of 3.32±0.48 levels for each patient. Among them, ACR was performed in 52 levels (62.6%). In the second-stage, TLIF at L5/S1 level was performed in 7 patients. The average lumbar interbody fusion levels were 3.60±0.82. The average posterior fixation segments were 6.20±1.78 in the staged group. PCO was performed in 9 patients (36%).

For the control group, 58 levels of TLIF were performed for all the patients. The average lumbar interbody fusion levels were 2.90±0.79, which showed significant difference when compared to staged group (*P*<0.01). The average posterior fixation segments were 8.25±1.16, which showed significant difference when compared to staged group (*P* < 0.01). Osteotomy was performed in 15 patients (75%), including PCO in 11 cases and PSO in 4 cases, respectively. There was significant difference between the two groups regarding the incidence of osteotomy (*P* < 0.01).

Compared to the control group, longer operative time was needed in the staged group. However, both blood loss and blood transfusion were significantly reduced in the staged group when compared to control group (Table 2). There was no significant difference between the two groups in postoperative hospital stays.

**Table 2:** Comparison of surgical results between staged group and control group

	Staged group			Control group	P value
	First-stage	Second-stage	Overall		
Operative time (min)	95.7±10.09	200.9±38.2	296.6±34.5	240.2±31.3	< 0.01
Blood loss (ml)	62.2±18.8	542±214.4	604.2±218.4	870±257.7	< 0.01
Blood transfusion (ml)	0	164±215.8	164±215.8	435±320	< 0.01
Postoperative hospital stays (day)	5.6±0.65	9.08±2.83	14.68±2.88	14.4±3.52	0.77

### Clinical results

Regarding to the preoperative ODI, VAS back and VAS leg, no significant difference was found between the two groups (Table 3). After surgery, significant improvements of ODI, VAS back and VAS leg were achieved in both groups (*P* < 0.01), and the improvements were maintained till to the final follow-up visit (*P* < 0.01). No difference in clinical improvement was found between the two groups postoperatively and at the final follow-up.

**Table 3:** Comparison of clinical results between staged group and control group

	Staged group	Control group	P value
Preoperative			
VAS (back)	6.3±1.4	6.6±1.5	0.61
VAS (leg)	6.1±1.1	5.9±0.9	0.54
ODI (%)	67.4±13.7	68.7±14.7	0.84
Postoperative			
VAS (back)	2.5±0.8	2.4±0.7	0.46
VAS (leg)	2.3±0.6	2.5±0.8	0.38
ODI (%)	39.5±10.5	42.3±11.4	0.09
Final follow-up			
VAS (back)	2.3±0.6	2.2±0.8	0.45
VAS (leg)	1.9±0.8	1.8±0.7	0.37
ODI (%)	26.8±8.8	29.3±9.2	0.19

### Radiological results

There were no significant differences between the two groups regarding to preoperative Cobb angle, CBD, SVA, LL, PT, PI and PI/LL mismatch (Table 4). Postoperative spinopelvic parameters were significantly improved in both groups (*P* < 0.01). Except Cobb angle (*P* = 0.04), no significant difference was found between the two groups regarding to postoperative CBD, SVA, LL, PT, and PI/LL mismatch. At the final follow-up, significant radiological improvements were maintained in both groups (*P* < 0.01), and no significant difference was found between the two groups.

**Table 4:** Comparison of spinopelvic parameters between staged group and control group

Preoperative	Staged group	Control group	P value
Preoperative			
Cobb(°)	38.2±4.67	39.75±5.07	0.31
CBD(cm)	4.68±1.09	4.25±1.16	0.21
SVA(cm)	7.77±1.15	7.46±1.03	0.34
LL(°)	11.36±11.71	12.25±11.82	0.81
PT(°)	29.48±4.4	32.3±6.06	0.08
PI(°)	49.4±4.9	49.6±5.1	0.87
PI/LL(°)	38±12.08	37.35±12.6	0.86
Postoperative			
Cobb(°)	11.0±3.76	13.9±5.4	0.04
CBD(cm)	1.8±0.62	1.6±0.59	0.28
SVA(cm)	2.85±0.97	2.57±1.06	0.36
LL(°)	42.44±6.08	43.05±6.03	0.74
PT(°)	16.68±3.39	18.7±4.23	0.08
PI/LL(°)	6.9±8.0	6.55±7.86	0.88
Final follow-up			
Cobb(°)	13.2±4.4	15.9±0.55	0.07
CBD(cm)	1.9±0.67	1.7±0.57	0.43
SVA(cm)	3.5±1.02	3.08±1.22	0.19
LL(°)	37.6±8.25	38.1±6.64	0.84
PT(°)	18.44±3.37	19.46±4.45	0.39
PI/LL(°)	11.72±9.42	11.5±7.88	0.93

### Complication

In the staged group, 11 complications occurred in 8 patients (32%), including 7 minor and 4 major complications. Major complications were as follows: one case of deep wound infection treated with debridement; one case of deep vein embolism treated with inferior vena cava mesh placement; one case of pulmonary infection treated with antibiotics; one patient developed PJK 16 months after surgery and was treated conservatively.

In the control group, 11 complications occurred in 9 patients (45%), including 6 minor and 5 major complications (*P* = 0.37). Major complications were as follows: one case of deep wound infection treated with debridement; one case of deep vein embolism was treated with anticoagulant; one case of cerebral infarction was treated with medication and rehabilitation exercise; one case of permanent lower extremity radiculopathy; one patient developed PJK 20 months after surgery and underwent revision surgery. All patients with surgical-related complication in both groups achieved great prognosis after timely and effective treatment.

## Discussion

This retrospective study was conducted to evaluate the clinical and radiological outcomes of first-stage LLIF in combination with second-stage PIF in the treatment of ADLS with sagittal imbalance, and to compare the treatment outcomes with surgical strategy of single-stage PIF alone. The results in our study indicated both corrective treatments were effective in reducing pain, improving function, and correcting coronal and sagittal deformities. However, staged LLIF in combination with PIF had advantages in reducing surgical trauma, fixation segments and osteotomy requirement, and had a tendency to reduce complications.

There is no definite surgical management algorithm for severe ADLS, which necessitates meticulous planning [16]. Except neural element decompression, it is essential to correct regional and global coronal and sagittal imbalances during surgical treatment. Sagittal balance, in particular, has been widely reported to be closely related to postoperative quality of life, instrumentation failure and adjacent segmental disease [17–20]. Traditionally, long-segment PIF with or without appropriate osteotomy was primary treatment for ADLS with sagittal imbalance. However, it is often associated with major surgical trauma and high potential complications [21]. Matsumura et al. [22] reported posterior corrective surgery using multilevel TLIF with rod rotation could provide effective deformity correction in coronal plane, while this procedure had limited potential in the correction of the deformity in the sagittal plane. The difficulty of inserting a larger interbody cage through a small portal to disc space during TLIF procedure may be one of the reasons for unsatisfactory correction of sagittal balance.

Osteotomy procedure is an effective option of management for correcting inflexible sagittal deformity. PCO, including Ponte osteotomy and Smith-Petersen osteotomy (SPO), can only provide 5°-10° of segmental sagittal correction [23]. Thus, PCO may be insufficient for severe sagittal deformity. In addition, anterior column mobility is a prerequisite for performing this kind of spinal osteotomy. PSO, as a three-column osteotomy procedure, is a transpedicular vertebral wedge resection, which can provide 25°-35° sagittal correction at a given level without anterior column lengthening. However, PSO procedure is often associated with major bleeding, neurological deficits, instrumentation failure and pseudarthrosis [4, 24]. Maida et al.[4] analyzed the complications related to osteotomy in the patients with severe rigid ADLS. They reported that the incidence of complications was 16.9% for SPO, while it reached to 46.2% for PSO.

In recent years, LLIF combined with PIF have been developed with good clinical results for the treatment of ADLS. Bae et al. [25] reported that compared with posterior spinal fusion alone or anterior lumbar interbody fusion combined with posterior spinal fusion, LLIF combined with posterior spinal fusion for adult spinal deformity (ASD) showed lower rates of PJK and mechanical failure at the upper instrumented vertebra and better clinical results. The ability of LLIF in the correction of coronal deformity has been proved previously. However, its effectiveness in restoring sagittal balance remains controversial. Sembrano et al. [26] reported that although LLIF could improve the intervertebral height and segmental lordosis, it could not effectively improve the overall LL, even using lordotic interbody cages. Acosta et al. [10] also reported the inadequacy of LLIF in improving regional and global sagittal alignment in patients with degenerative lumbar disease. Some reports indicated that postoperative sagittal alignment was influenced by the number of LLIF levels [27, 28]. However, in the studies of Sembrano et al. and Acosta et al. [10, 26], the number of LLIF levels per patient was only one or two-levels. We previously reported that multilevel LLIF (3.2 levels per patient) combined with ACR at the first-stage could decrease the Lenke-Sliva classification grading, and subsequently simplified posterior surgical procedure at the second-stage [27]. Similarly, Park et al. [29] reported that the average correction of segmental lordosis provided by one ACR was about 14.9°, which was not influenced by the disc space collapse and segmental stiffness. ACR has been developed as a minimally invasive alternative to three-column osteotomy for sagittal imbalance correction. In the current study, by using multilevel LLIF (3.32 levels per patient), ACR (62.6% of the LLIF levels) and PIF, the sagittal parameters including SVA, LL and PI-LL mismatch were significantly improved after surgery and well maintained during the follow-up period. Compared with control group, staged group showed less surgical trauma, fewer posterior fixation segments and lower osteotomy requirements.

The purpose of staged treatment for severe ADLS is to reduce surgical invasiveness of each procedure. However, it may also prolong hospital stay and bed rest, leading to the development of related complications. Passias et al. [11] reported that staged spinal fusion for ASD, which added ALIF and LLIF to the procedure, resulted in significantly higher incidence of peri- and postoperative complications leading to revision compared with single-stage procedure. Arzeno et al. [30] reported there was no difference in infection rate between staged procedure and sing-stage procedure, but thrombotic events increased significantly. However, Yamato et al. [31] reported two-stage or single-stage treatment showed similar complication incidence in patients with ADS, and two-stage treatment even provided better spinal deformity correction and clinical results with less early reoperation. Similar in our study, there was no significant difference in the incidence of complications between the two groups, and staged group even showed a tendency to reduce the total complication incidence. The underlying reasons maybe that LLIF was a minimally invasive procedure. Patients in the staged group were encouraged to ambulate on the first day after the first-stage surgery, and the interval between the first- and second-stage procedure was relatively short.

There are several limitations to the current study. Retrospective analysis reduced the evidence level. A small sample size was another weak point, limiting its statistical power. Further study is needed with a larger cohort included. Finally, the follow-up period is relatively short. A prolonged observation period is beneficial in the comparison of the efficacy of staged LLIF combined with PSF and PSF alone in the treatment of ADLS. Hence, a high-quality randomized controlled study of with larger sample size and longer follow-up duration is required to confirm our finding in the future.

## Conclusion

Both staged LLIF combined with PIF and PIF alone were effective treatments for ADLS with sagittal imbalance. However, staged treatment showed less surgical trauma, which reduced the number of posterior fixation segments and osteotomy requirement.

## Data Availability

The datasets used and/or analyzed during the current study are available from the corresponding author on reasonable request.
